# Enhancement of Paramagnetic Relaxation by Photoexcited Gold Nanorods

**DOI:** 10.1038/srep24101

**Published:** 2016-04-13

**Authors:** Tao Wen, Wayne G. Wamer, Witold K. Subczynski, Shuai Hou, Xiaochun Wu, Jun-Jie Yin

**Affiliations:** 1 Division of Analytical Chemistry, Office of Regulatory Science, Center for Food Safety and Applied Nutrition, US Food and Drug Administration, College Park 20740, MD, USA; 2 CAS Key Laboratory of Standardization and Measurement for Nanotechnology, National Center for Nanoscience and Technology, Beijing 100190, P. R. China; 3 Institute of Basic Medical Sciences, Chinese Academy of Medical Sciences & Peking Union Medical College, Beijing 100005, P. R. China; 4 Division of Bioanalytical Chemistry, Office of Regulatory Science, Center for Food Safety and Applied Nutrition, US Food and Drug Administration, College Park 20740 MD, USA; 5 Department of Biophysics, Medical College of Wisconsin, Milwaukee 53226 WI, USA

## Abstract

Electron spin resonance (ESR) spectroscopy was used to investigate the switchable, light-dependent effects of gold nanorods (GNRs) on paramagnetic properties of nitroxide spin probes. The photoexcited GNRs enhanced the spin-spin and spin-lattice relaxations of nitroxide spin probes. It was shown that molecular oxygen plays the key role in this process. Our results demonstrate that ESR is a powerful tool for investigating the events following photoexcitation of GNRs. The novel light-controlled effects observed for GNRs on paramagnetic properties and activities of surrounding molecules have a number of significant applications where oxygen sensing and oxygen activity is important.

Localized surface plasmon resonance (SPR)^1^,^2^ is an novel and useful optical property of noble metal nanoparticles. Because the localized SPR properties of gold nanorods (GNRs) are easily tuned by changing their size, shape, structure and surrounding dielectric environment, GNRs have a number of growing chemical^3^,^4^ and biomedical^3^,^4^,^5^ applications^2^. While much is known about the photophysical and photochemical events following excitation of GNRs, much remains to be learned about the interactions between photoexcited GNRs and their physical and chemical environment. Here we use electron spin resonance (ESR) spectroscopy to investigate alterations in paramagnetic interactions triggered by photoexcitation of GNRs. ESR is a direct and reliable method to identify and quantify unpaired electrons (i.e. paramagnetic species) in liquid, solid and even gas samples in both chemical and biological environments. ESR techniques are, beyond any doubt, the reference techniques for detection and characterization of paramagnetic species^6^. In addition, experiments using ESR can be designed to unravel interactions of paramagnetic species with their chemical or biochemical environments.

Here we employed ESR to investigate the switchable, light-dependent effects of GNRs on paramagnetic properties of molecules in the surrounding environment. Firstly, in these investigations we used two stable nitroxide free radicals, namely ^15^N-PDT (4-oxo-2,2,6,6-tetramethylpiperidine-d_16_-1-oxyl) or CTPO (3-carbamoyl-2,5-dihydro-2,2,5,5-tetramethyl-1H-pyrrol-1-yloxyl), as paramagnetic probes. We found that photoexcited GNRs influenced the spin relaxation of these spin probes which was measured as the altered line widths (LWs) of ^15^N-PDT or the loss of super-hyperfine structure of CTPO in their ESR spectra. Photoexcited GNRs also influenced the microwave power at which the ESR signal of spin probe was saturated. In further investigations, molecular oxygen was found to play a key role in the effect of photoexcited GNRs on spin relaxation of the spin probes. This is the first report of the novel light-control effects of GNRs on paramagnetic properties of surrounding molecules. In addition, the described approach will be valuable for further investigation of the properties of plasmonic nanomaterials.

## Results

**Figure 1:**
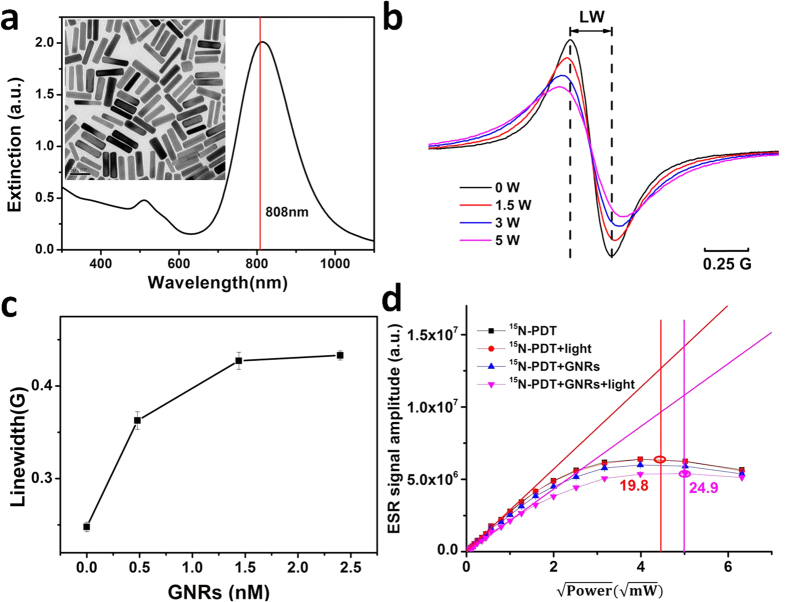
Effects of photoexcited GNRs on relaxation times. (**a**) UV-vis-NIR extinction spectrum of CTAB coated GNRs dispersed in water. Inset is a typical transmission electron microscopy (TEM) image of the prepared GNRs. (**b**) ESR spectra of 100 μM ^15^N-PDT and 2.4 nM GNRs exposed to different powers of the 808 nm laser for 10 min. The LWs are 0.27, 0.32, 0.39, 0.43 G for 0, 1.5, 3, 5 W, respectively. (**c**) LWs of ESR spectra for samples having different concentrations of GNRs and 100 μM ^15^N-PDT during irradiation with an 808 nm laser (5 W) for 10 min. (**d**) Microwave power saturation curve for 100 μM ^15^N-PDT in the absence and presence of 2.4 nM GNRs at 37 °C. Samples were in the dark or irradiated with an 808 nm laser (5 W). The saturating (P_1/2_) power is shown as circle indicated. Note that the molar concentration of GNRs here means the concentration of nanoparticles (See Supplementary Information). The power in the (b) indicates the power of laser beam, while the power in the (d) indicates the microwave power of the ESR spectrometer.

The UV-vis-NIR extinction spectrum of cetyltrimethylammonium bromide (CTAB) coated GNRs in water has two extinction maxima at 511 nm and 810 nm, resulting from exciting the GNR’s transverse and longitudinal surface plasmons, respectively (Fig. 1a). These CTAB coated GNRs have an average length of 60.6 ± 7.5 nm and an aspect ratio of 4 (Fig. 1a, insert). Changes in the ESR spectra of ^15^N-PDT sensitively report changes in paramagnetic interactions with their chemical environment. These spectral changes are induced by factors which alter the rate of collisions or strength of interactions between ^15^N-PDT and other paramagnetic molecules. The value of ^15^N-PDT as a spin probe is evident from its many applications, including its use in ESR oximetry for measuring levels of molecular oxygen. Here, ^15^N-PDT was used to probe the effects of photoexcited GNRs on chemical species in the surrounding medium. We observed that the LW of ^15^N-PDT significantly increased during photoexcitation of the GNR’s longitudinal SPR using an 808 nm laser (Fig. 1b). The LW of ^15^N-PDT in the suspension of GNRs increased from 0.26 ± 0.01 G to 0.43 ± 0.01 G with 808 nm laser (5 W) irradiation, and was dependent on the power of the laser. Effects on the LW of ^15^N-PDT were also closely dependent on the concentration of GNRs (Fig. 1c) and their extinction spectrum (Supplementary Fig. 1), indicating the role of GNRs’ SPR on the LW of ^15^N-PDT. Another spin probe (CTPO) was used to confirm the generality of the observed effect. We demonstrated that photoexcitation of GNRs results in the reduction in the super hyperfine structure of CPTO and dependence on the power of the incident laser radiation (Supplementary Fig. 2 and Supplementary Fig. 3).

The broadening of ^15^N-PDT’s LW and reduction in super hyperfine structure of CTPO result from shortening the electron spin relaxation times for these spin probes^7^,^8^. As an additional demonstration of these effects on spin relaxation times, we examined the effects of photoexcited GNRs on the microwave power saturation of the ESR signal for ^15^N-PDT^9^. In power saturation experiments, shortening of the spin-lattice relaxation time (T_1_) and/or the spin-spin relaxation time (T_2_) is exhibited by an increase in the microwave power needed to saturate the ESR signal. We determined that P_1/2_ for ^15^N-PDT, which is the microwave power at which the signal is half as great as it would be in the absence of saturation, increased from 19.8 mW to 24.9 mW in the presence of photoexcited GNRs (Fig. 1d). We can summarize that both the broadening of ^15^N-PDT’s LW and a higher saturation power indicate that photoexcited GNRs shorten the spin relaxation time of the spin probe.

**Figure 2:**
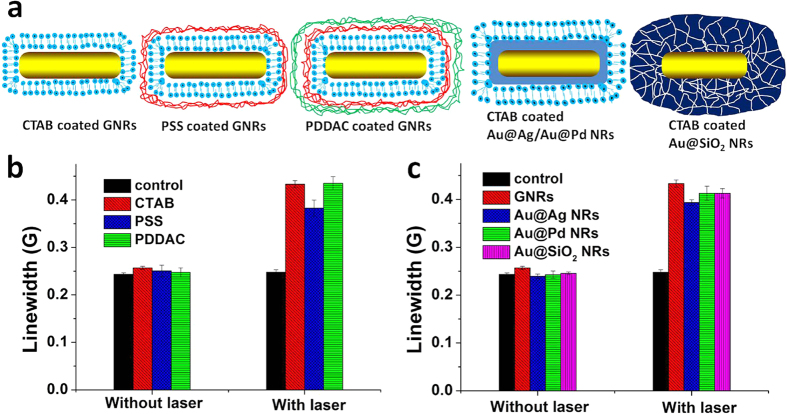
Effects of various surface-coatings of GNRs. (**a**) Schematic of different surface coatings of GNRs and bimetallic core-shell structures based on GNRs core. (**b**,**c**) LWs of ESR spectra without and with 808 nm laser irradiation (5 W) for 10 min, the solutions containing 100 μM ^15^N-PDT and 2.4 nM GNRs with different (**b**) surface coatings or (**c**) core-shell structures. The control includes only 100 μM ^15^N-PDT.

To investigate the effect of the surface of GNRs on the observed phenomena, GNRs with six different surface alterations were synthesized. Schematics and typical TEM images of GNRs with different coatings (positive charge: CTAB, poly(diallyldimethyl ammonium chloride) (PDDAC) and negative charge: poly(sodium-p-styrenesulfate (PSS)) and different components (metal: Ag, Pd and non-metal: mesoporous SiO_2_) are shown in Fig. 2a and Supplementary Fig. 4. When these surface-modified GNRs were photoexcited at 808 nm, the LW of ^15^N-PDT increased similarly for all coated GNRs (Fig. 2b,c). This result demonstrates that the SPR-driven effect of GNRs on the LW of ^15^N-PDT is independent of surface alterations including changes in surface coating and surface composition. These results suggest that direct interactions at the surface of GNRs with either the spin probe or other species are unnecessary for eliciting the light-driven effects of GNRs on the LW of ^15^N-PDT. The primary structural requirement for the observed effects is a GNR core with SPR excitable by the light source.

**Figure 3:**
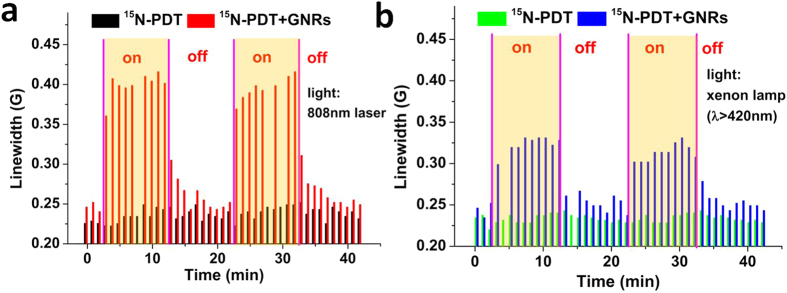
Controlled reversibility. LWs of ESR spectra of 100 μM ^15^N-PDT in the absence and presence of 2.4 nM GNRs excited with a 5 W 808 nm laser or a xenon lamp (λ > 420 nm). The shaded areas indicate time intervals during irradiation.

With exposure to on-off cycles of laser irradiation, we observed that the line broadening effects of photoexcited GNRs has cyclic reversibility (Fig. 3a). The LW of the ^15^N-PDT ESR signal increases rapidly upon excitation with the 808 nm laser (5 W) and this increase is maintained throughout irradiation. After the end of irradiation, the LW returns rapidly to the pre-irradiation magnitude. This pattern persists through several on-off cycles of irradiation. Figure 3b shows a similar cyclical reversibility for LW broadening when using a xenon lamp filtered to emit at wavelengths greater than 420 nm, indicating a general role of light on GNRs. Due to the differences in the intensity at 810 nm, irradiation with the xenon light resulted in the smaller increases in LW. This cyclical reversibility property observed for noble metal nanorods allow them to be potentially used for light activated switching and time resolution of molecular events.

**Figure 4:**
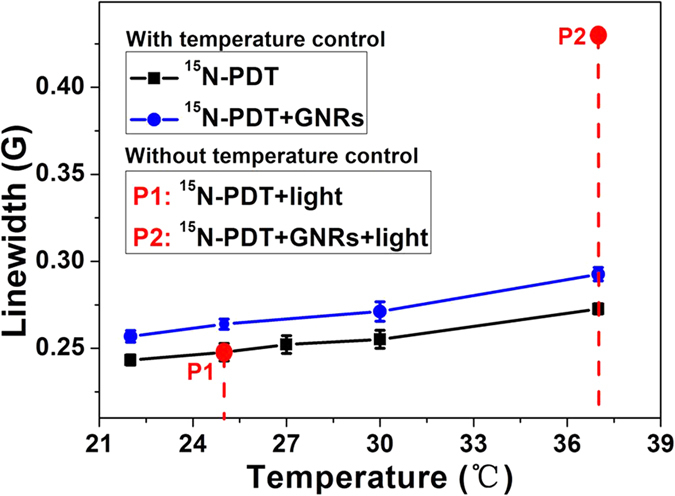
Temperature influence on relaxation times. Temperature effects on LWs of 100 μM ^15^N-PDT and 2.4 nM GNRs with thermostatically varied and no irradiation, or with 808 nm laser irradiation (5 W) for 10 min without temperature control.

It is well known that the part of the energy absorbed during photoexcitation of GNRs is converted to heat^10^. Increased temperature caused by this photothermal effect could result in a broadened LW for the spin probe by increasing the frequency of collisions between spin probe molecules (Fig. 4)^11^. We measured the changes in temperature during irradiation in both the presence and absence of GNRs (Supplementary Fig. 5). During irradiation with an 808 nm laser (5 W), the temperatures of solutions in the absence and presence of GNRs increased from 22 °C to 25 °C and 37 °C, respectively. We therefore examined whether photothermal effects underlie the observed broadening of ^15^N-PDT’s LW. We observed that a much larger increase in LW was noted for photoexcited GNRs than for unirradiated GNRs at the same temperature (37 °C, Fig. 4). These results demonstrate that the effect of photoexcited GNRs on ^15^N-PDT’s LW cannot be solely attributed to a macroscopic rise in temperature and increased collision rates between paramagnetic species.

**Figure 5:**
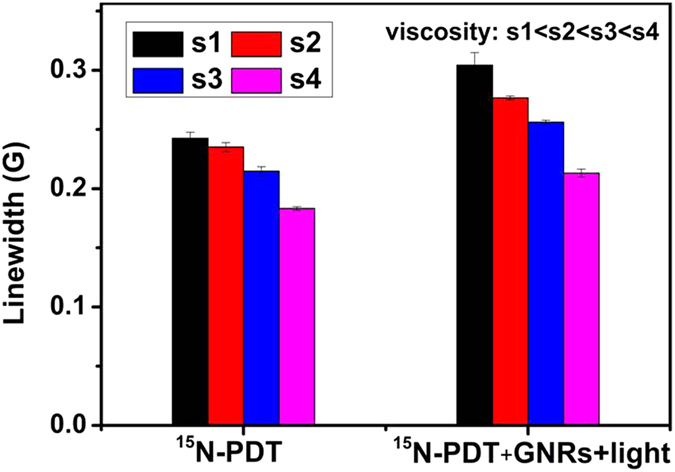
LW changes for 100 μM ^15^N-PDT in the absence and presence of 2.4 nM CTAB coated GNRs with xenon lamp irradiation (λ > 420 nm) for 10 min in solutions with different viscosities (viscosity: s1 < s2 < s3 < s4. Solutions of s1, s2, s3, and s4 contain 0%, 10%, 19%, and 35% glycerol, respectively).

The change of LWs observed above clearly demonstrate that photoexcitation of GNRs results in shortened electron spin relaxations times for ^15^N-PDT which can be seen as increases in ^15^N-PDT’s LW. However, these results do not reveal the nature of the interactions leading to the SPR-driven effects of GNRs on ^15^N-PDT’s spin relaxation times. Paramagnetic relaxation can result from two types of interactions, dipole-dipole and Heisenberg exchange^9^,^12^. The relative importance of dipole-dipole and Heisenberg exchange can be determined by examining how the viscosity of the medium effects increases in ^15^N-PDT’s LW in the presence of photoexcited GNRs^13^. Dipole-dipole interactions are averaged out to zero in the fast-tumbling systems expected at low viscosity. Exchange interactions, on the other hand, usually proceed through collisions of spin-bearing functionalities and thus are mostly observed in low viscosity, fast-tumbling systems. Therefore, if the effects of photoexcited GNRs on the LW of ^15^N-PDT result from exchange interactions, larger changes in the LW are expected at lower viscosities. Similar to superparamagnetic Fe_2_O_3_ nanoparticles, which affect the spin exchange interactions^14^,^15^, we observe that the effects of photoexcited GNRs on the LW of ^15^N-PDT decrease with increasing viscosity (Fig. 5). Therefore, the effects of photoexcited GNRs on the LW of ^15^N-PDT can be attributed to Heisenberg exchange involving spin exchange between ^15^N-PDT molecules and/or spin exchange between ^15^N-PDT and other paramagnetic species.

**Figure 6:**
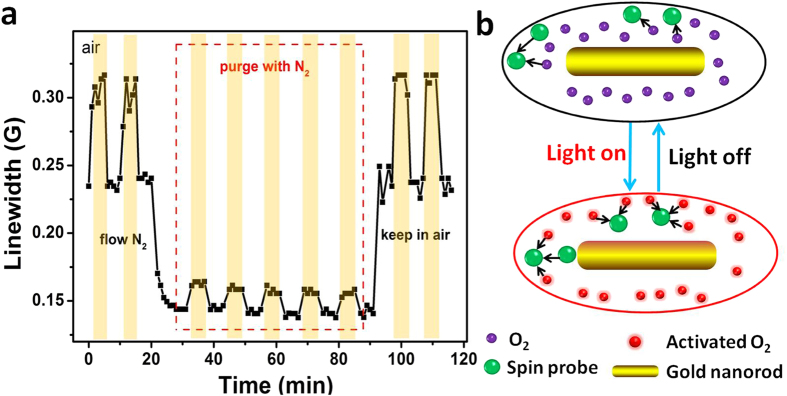
Effects of molecular oxygen. (**a**) Oxygen dependence and cyclicity of LW changes for 100 μM ^15^N-PDT and 2.4 nM GNRs contained in a TPX capillary with on/off cycling of 808 nm laser irradiation (5 W). Following two cycles of exposure of air saturated solutions, samples were purged with N_2_, and then back to air saturated solutions. The shadows show that the light is on. (**b**) Schematic for oxygen activation around the photoexcited nanorod.

These viscosity effects demonstrate that photoexcited GNRs cause broadening of ^15^N-PDT’s LW predominately through Heisenberg spin exchange interactions. However, the spin system(s) interacting with ^15^N-PDT are not identified. Spin exchange interactions could result from direct interaction of the spin probe with GNRs or interactions with another paramagnetic species. It is well established that molecular oxygen, which in the electronic ground state (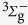) is paramagnetic, broadens the LW of spin probes through Heisenberg exchange interactions. Indeed, this is the basis for the use of ESR to measure concentrations of dissolved oxygen (ESR oximetry)^7^. To examine the role of dissolved oxygen, we compared the effects of photoexcited GNRs on ^15^N-PDT’s LW in air saturated solutions and in solutions purged with nitrogen (N_2_). As shown in Fig. 6, we observed that purging with N_2_ greatly reduces the effects of photoexcited GNRs on ^15^N-PDT’s LW without altering the on/off cycling associated with photoexcitation. The effects of purging with N_2_ were reversible. Equilibration of samples back to air, resulted in a return to the initially observed effects under air saturated conditions. Compared to LWs for unirradiated GNRs, LWs with photoexcited GNRs increased 31% in the air and only 12.5% after purging with N_2_. These results indicate that dissolved oxygen is involved in the SPR-driven effects of GNRs on the LW of ^15^N-PDT and that these effects were reversible. Similar reversibility by purging with N_2_ were observed using CTPO as a spin probe (Supplementary Fig. 6).

We examined several possible mechanisms for oxygen’s role in shortening the spin probe’s relaxation time and increasing its LW in the presence of photoexcited GNRs. One possible mechanism is release of oxygen, adsorbed to the surface of the GNRs, during photoexcitation. The increase in oxygen concentration resulting from oxygen desorption from the surface of photoexcited GNRs would result in an increased frequency of collisions between the spin probe and oxygen, thus enhancing Heisenberg spin exchange. However, it is known that, except for some clusters of gold smaller than 22 atoms, gold surfaces do not appreciably adsorb molecular oxygen^16^,^17^. In addition, the magnitude of the effects of photoexcited GNRs on the LW of ^15^N-PDT makes release of adsorbed oxygen a very unlikely mechanism. We have determined that an increase of 175.3 μM oxygen would be needed to produce the observed LW broadening of ^15^N-PDT from 0.26 G (22 °C) to 0.43 G (37 °C) (Supplementary Fig. 7). Another possible mechanism involves formation of a highly paramagnetic oxygen species in the presence of photoexcited GNRs. Several investigators have reported that reactive oxygen species (ROSs), including superoxide radical and singlet oxygen, are detected following excitation of the SPR of GNRs^18^,^19^,^20^,^21^. Some of these ROSs, such as superoxide radical, are paramagnetic. Using ESR with spin trapping and selective quenchers of superoxide radical and singlet oxygen, we found no evidence that either superoxide radical or singlet oxygen was formed in our experiment conditions (Supplementary Fig. 8). In addition, quenchers of superoxide radical or singlet oxygen did not reduce the effects on LW broadening induced by photoexcitation of GNRs (Supplementary Fig. 9). These results indicate that molecular oxygen, rather than ROSs, plays an important role in the effects of photoexcited GNRs on the spin relaxation of ^15^N-PDT.

The role played by oxygen may suggest a mechanism through which photoexcited GNRs enhance relaxation of spin probes. Because relaxation of spin probes is independent of surface coatings, direct interactions between the surface of GNRs and oxygen or other species affecting paramagnetic relaxation may be viewed as unimportant. This is consistent with our observations that electron transfer, to produce new paramagnetic species, is absent during photoexcitation of GNRs. We have also observed that changes in the macroscopic temperature of samples during irradiation of GNRs do not fully account for the observed light-driven effects of GNRs on paramagnetic relaxation time. Investigators have observed that temperatures measured distant (millimeter scale) from photoexcited metal nanomaterials are significantly lower than temperatures near (nanometer scale) the surface of photoexcited metal nanomaterials^10^. We propose that this non-thermalized temperature distribution results in increased motion of oxygen located near photoexcited GNRs. The resultant increase in collisions between oxygen and the spin probe would explain increased paramagnetic relaxation times for the spin probe through Heisenberg spin exchange. In short, GNRs are locally heated in the presence of light and the diffusion of all the molecules around the GNRs increase. This phenomenon is reflected in LW increasing (shortening of relaxation times physically).

## Discussion

For the first time, we have observed that GNRs, irradiated to excite their SPR, can decrease the spin relaxation time of ^15^N-PDT through a Heisenberg spin exchange mechanism. Our results indicate that molecular oxygen plays a major role in the effects of photoexcited GNRs on the spin relaxation of ^15^N-PDT. The experimental approach which we describe will be broadly applied to other plasmonic nanoparticles for investigating interactions of photoexcited nanoparticle with their environments. In addition, the results suggest new applications for photoexcited GNRs. An important application is in medical imaging. To illustrate the potential utility of photoexcited GNRs for medical imaging, we have used the described ESR techniques to examine the effects of the MRI contrast agents, Fe_2_O_3_ and gadopentate, on the spin relaxation of ^15^N-PDT (Supplementary Fig. 11). Effects on spin relaxation times analogous to those seen with photoexcited GNRs were seen for these two well established contrast agents. Advantages to use of photoexited GNRs in medical imaging include increased control of spatial and temporal resolution during irradiation. Additional significant potential applications include ESR imaging and ESR microscopy and photoswitchable contrast imaging^22^,^23^,^24^,^25^. For these applications, GNRs have particular advantages. Firstly, GNRs are photoexcited at wavelengths of light which are transmitted through most biological tissues. In addition, spatial and temporal resolution of images can be varied by changing the field and timing of illumination. Also, our results indicate that effects on spin relaxation times can be obtained under conditions which do not result in significant heating. Finally, the sensitivity of the observed effects of photoexcited GNRs on oxygen would allow measurement of oxygen partial pressures in cells or tissues. Due to the influence of ROS (related to oxygen) on tissues, this would be particularly advantageous for differentiating malignant from normal tissues which differ in their usage of oxygen. Therefore, this novel phenomenon we observed has significant potential biomedical applications.

## Methods

### Chemicals

Sodium borohydride (NaBH_4_), chlorauric acid (HAuCl_4_·3H_2_O), cetyltrimethylammonium bromide (CTAB), sodium citrate, silver nitrate (AgNO_3_), L-ascorbic acid (AA), poly(sodium-p-styrenesulfate) (PSS, molecular weight: 70,000), poly(diallyldimethyl ammonium chloride) (PDDAC, 20%), and potassium tetrachloropalladate(II) (K_2_PdCl_4_) were purchased from Alfa Aesar and used as received. 4-Oxo-2,2,6,6-tetramethylpiperidine-d_16_-1-oxyl (^15^N-PDT) was purchased from Cambridge Isotope Labs (Andover, MA). 5-tert-Butoxycarbonyl-5-methyl-1-pyrroline N-oxide (BMPO) was purchased from Bioanalytical Labs (Sarasota, FL). Dimeglumine gadopentetate injection (Gds) is from BEILU Pharmaceutical Co., Ltd (Beijing, China). 3-Carbamoyl-2,5-dihydro-2,2,5,5-tetramethyl-1H-pyrrol-1-yloxyl (CTPO), 2,2,6,6-tetramethyl-4-piperidine (TEMP), sodium chloride (NaCl), sulfuric acid (H_2_SO_4_), tetraethyl orthosilicate (TEOS), sodium hydrate (NaOH), superoxide dismutase (SOD), and sodium azide (NaN_3_) were all purchased from Sigma-Aldrich (St. Louis, MO). Milli-Q water (18 MΩ cm) was used for preparation of all solutions.

### Typical Synthesis of GNRs

GNRs were synthesized using a standard seed-mediated growth method. Briefly, CTAB-capped Au seeds: CTAB (7.5 mL, 0.1 M) were diluted with water to 9.4 mL. Then, ice-cold NaBH_4_ (0.6 mL, 0.01 M) was added with continuous stirring for 3 min. The Au seeds were used within 2–5 h. For a typical preparation of the GNRs, a growth solution consisting of a mixture of CTAB (10 mL, 0.1 M), HAuCl_4_ (200 μL, 25 mM), AgNO_3_ (100 μL, 10 mM), H_2_SO_4_ (100 μL, 1 M), and AA (80 μL, 0.1 M) was added to the seed solution (24 μL) to initiate the growth of GNRs. During nanorod growth, the temperature was kept at 30 °C. After 12 h, the GNRs were purified by centrifuging the solution twice at 12,000 rpm for 5 min. The precipitate was collected and redispersed in Milli-Q water.

### Typical Synthesis of Au@Ag NRs, Au@Pd NRs and mesoporous Au@SiO_2_ NRs

There NRs were prepared as reported previously^26^,^27^,^28^ based on the above GNRs as the core.

### Polyelectrolyte-coated GNRs

The multilayer polyelectrolyte-coated GNRs were synthesized by sequentially coating negatively charged PSS and positively charged PDDAC onto the as-synthesized CTAB-coated GNRs^29^. For PSS coating, 10 mL 0.5 nM GNRs were centrifuged at 12,000 rpm for 10 min once, and the precipitate was dispersed in 10 mL of 2 mg/mL PSS aqueous solution (containing 6 mM NaCl). Then the solution was stirred for 3 h. After that, it was centrifuged at 12,000 rpm for 10 min, and the precipitate was redispersed in 10 mL water. For coating with PDDAC, a similar procedure was used.

#### Characterization of nanostructures

UV-vis-NIR extinction spectra were obtained using a Varian Cary 50 spectrophotometer. Transmission electron microscopy (TEM) images were captured on a FEI TECNAI G^2^ F20 U-TWIN at an accelerating voltage of 200 kV. The samples for TEM analysis were prepared by adding drops of the redispersed colloidal solutions onto standard holey carbon-coated copper grids, which were then air dried at room temperature. The GNR concentration is estimated using inductively coupled plasma mass spectrometer (ICP-MS, NexION300D) and TEM (The method used is described in the Supplementary information)^30^.

#### Electron spin resonance

All ESR measurements were carried out using a Bruker EMX ESR spectrometer (Billerica, MA) at ambient temperature. 50 μL aliquots of control or sample solutions (suspensions) were put in quartz capillary tubes with internal diameters of 0.9 mm and sealed. The capillary tubes were inserted into the ESR cavity and the spectra were recorded at selected times. Lasers emitting at 532 nm (3 W maximum) and 808 nm (5 W maximum) with a 5~8 mm diameter beam at the aperture were used in ESR studies. A 450 W xenon lamp coupled with a high pass filter was also used to provide light with wavelengths greater than 420 nm. Samples, in quartz capillary tubes, were irradiated directly in the ESR cavity during collection of ESR spectra.

In most ESR experiments, samples, commonly contained in quartz capillary tubes (except mentioned), were air saturated at ambient temperature, i.e., were open to the atmosphere until the capillary tubes were sealed to collect ESR spectra. In experiments conducted to examine the role of dissolved oxygen, samples were transferred to a 0.6 mm i.d. capillary made of gas-permeable methylpentene polymer (TPX) (Gift from Prof. J. S. Hyde, at the National Biomedical ESR Center, Medical College of Wisconsin) and used for ESR measurements^31^. Samples in the TPX capillary were purged with nitrogen or air for 10 min.

#### Collection of electron spin resonance spectra

For experiments with ^15^N-PDT (0.1 mM) ESR settings were as follows: 0.05 G field modulation, 2 G scan range, and 1 mW microwave power. All ESR recordings were performed using the low field line of ^15^N-PDT ESR spectra. For experiments with CTPO (0.1 mM) ESR settings were as follows: 0.04 G field modulation, 5 G scan range, and 1 mW microwave power. All ESR recordings were performed using the central line of CTPO ESR spectra.

LW is calculated from ESR signal as Fig. 1b shown. The standard deviations of LWs are obtained from at least three independent measurements.

BMPO and TEMP were employed to investigate the possible existence of superoxide radical and singlet oxygen, respectively. ESR settings were as follows: 1 G field modulation, 100 G scan range, and 20 mW microwave power.

#### Microwave power saturation experiment

In the microwave power saturation experiment, the peak-to-peak height of the ESR signal for ^15^N-PDT is measured as the microwave power is increased^9^. The power, P_1/2_, is the incident power at which the signal is half as great as it would be in the absence of saturation. P_1/2_ is a function of T_1_ and T_2_ of the nitroxide spin probe:


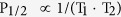


Effects on relaxation times are determined by plotting the signal amplitude as a function of the square root of the power, 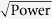. At low powers, when the equilibrium population of spin states remains unperturbed, the height of the signal increases in proportion to the square root of the power. A solid line (Fig. 1d, no saturation) extrapolating the initial slope shows the theoretical behavior in the absence of saturation. The Y axis of saturation power is half of the crossover point of this solid line and the line which has the same X value as saturation power.

## Additional Information

**How to cite this article**: Wen, T. *et al*. Enhancement of Paramagnetic Relaxation by Photoexcited Gold Nanorods. *Sci. Rep*. **6**, 24101; doi: 10.1038/srep24101 (2016).

## Supplementary Material

Supplementary Information
