# Supplementary material for: Enhancement of Paramagnetic Relaxation by Photoexcited Gold Nanorods
Source: Sci Rep. 2016 Apr 13;6:24101. doi: 10.1038/srep24101 (PMC4829829; doi:10.1038/srep24101)
Supplement: Supplementary Information [file srep24101-s1.doc]

**Supplementary information for:**

**Enhancement of Paramagnetic Relaxation by Photoexcited Gold Nanorods**

Tao Wen,^a, b, c^ Wayne G. Wamer,^d^ Witold K. Subczynski,^e^ Shuai Hou,^b^ Xiaochun Wu^*b^ and Jun-Jie Yin^*a^

^a^ Division of Analytical Chemistry, Office of Regulatory Science, Center for Food Safety and Applied Nutrition, US Food and Drug Administration, College Park 20740, MD, USA.

^b^ CAS Key Laboratory of Standardization and Measurement for Nanotechnology, National Center for Nanoscience and Technology, Beijing 100190, P. R. China.

^c^ Institute of Basic Medical Sciences, Chinese Academy of Medical Sciences & Peking Union Medical College, Beijing 100005, P. R. China.

^d^ Division of Bioanalytical Chemistry, Office of Regulatory Science, Center for Food Safety and Applied Nutrition, US Food and Drug Administration, College Park 20740 MD, USA.

^e^ Department of Biophysics, Medical College of Wisconsin, Milwaukee 53226 WI, USA.

*Corresponding author.

E-mail address: junjie.yin@fda.hhs.gov, wuxc@nanoctr.cn

**Supplementary Information**


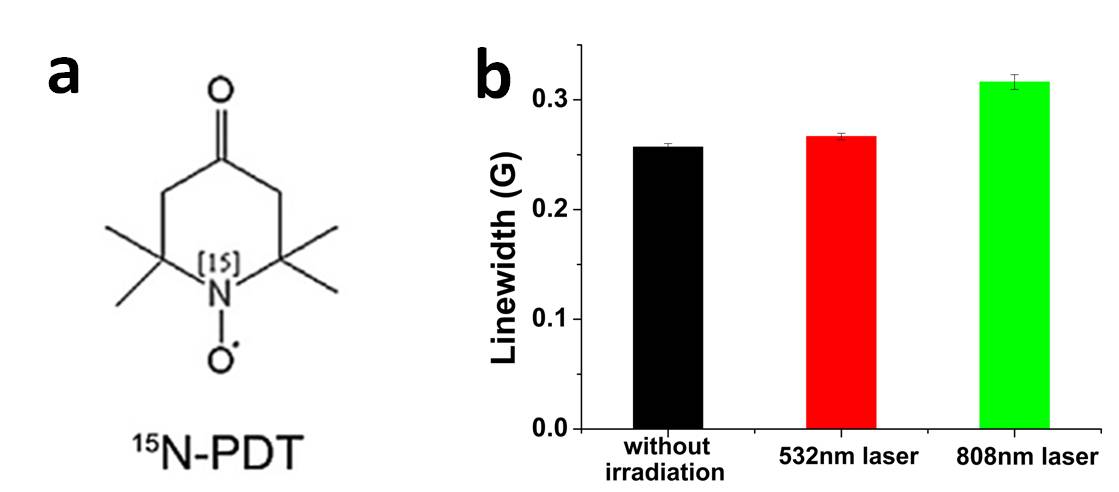


**Supplementary Figure 1.** (a) Chemical structure of the spin probe ^15^N-PDT and (b) LWs of ESR spectra for mixtures containing 100 μM ^15^N-PDT and 2.4 nM CTAB coated GNRs in the absence and presence of laser (3 W) irradiation for 10 min.

We observed that the line width (LW) for ^15^N-PDT in the presence of unirradiated nanorods or nanorods irradiated with a 532 nm laser (3 W) was 0.26 ± 0.01 G. In contrast, the LW of ^15^N-PDT increased to 0.32 ± 0.01 G during excitation near the longitudinal SPR using an 808 nm laser (3W) (Supplementary Fig. 1). This change is consistent with the absorption spectra of GNRs (A_808nm_>A_532nm_), which indicates that increases in ^15^N-PDT’s LW are due to absorption through the SPR. The molar concentrations given for GNRs are the particle concentrations.

**
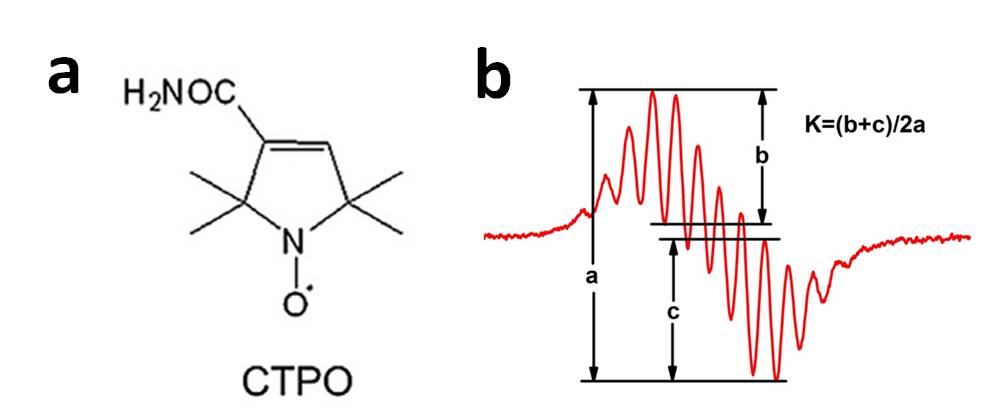
**

**Supplementary Figure 2.** (a) Chemical structure of the spin probe ^15^N-PDT and (b) definition of K parameter from the ESR spectra of the spin probe CTPO.


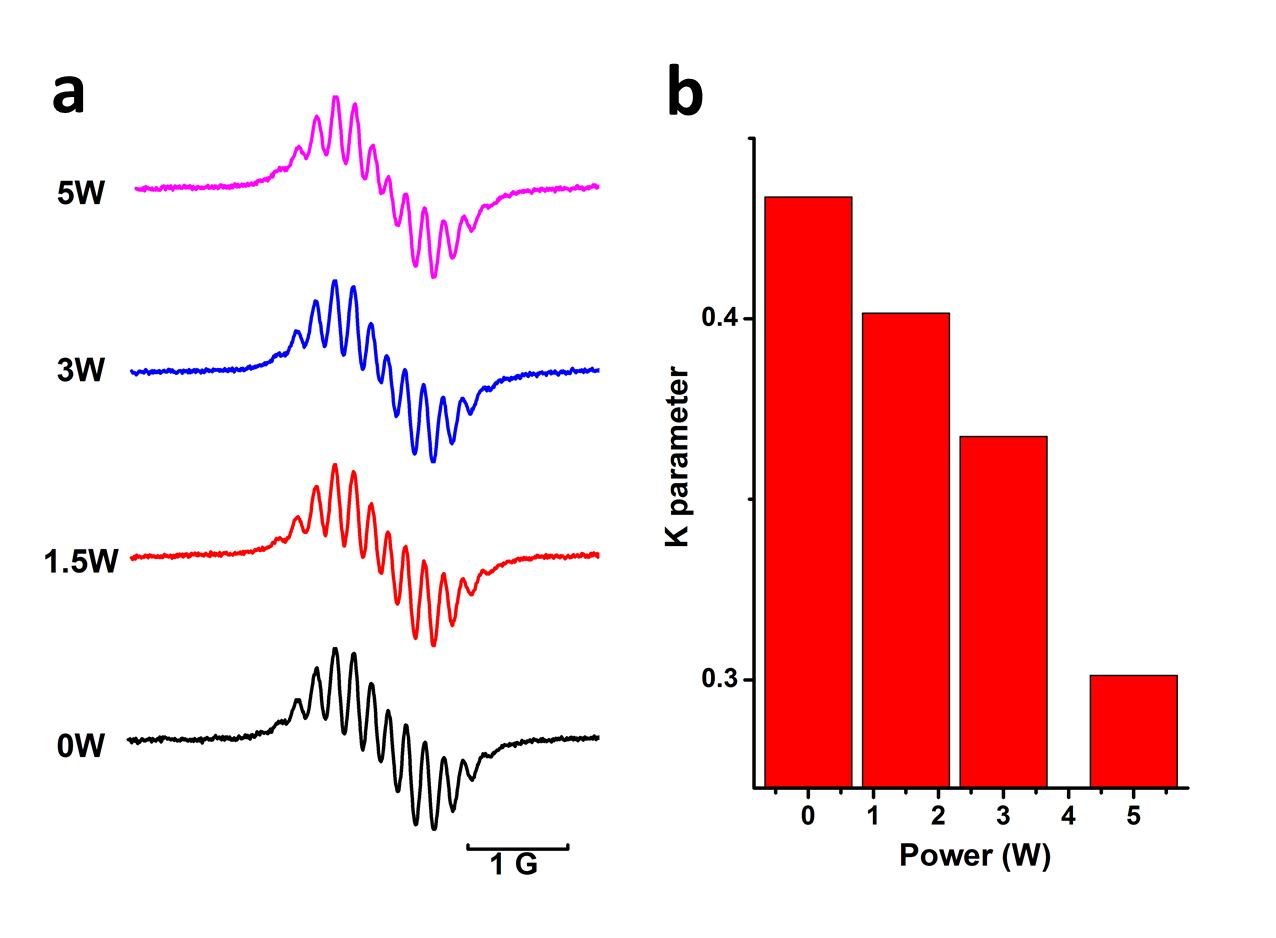


**Supplementary Figure 3.** ESR spectra and histograms of the K parameter for samples containing 100 μM CTPO and 2.4 nM GNRs exposed to different powers of an 808 nm laser for 10 min in a TPX capillary with purging with nitrogen.

ESR spectra of the spin probe CTPO exhibit three lines due to the hyperfine interaction of the unpaired electron with the spin of the nitrogen nucleus. Each line is further split into another group of lines because of super hyperfine interactions with spins of hydrogen nuclei. The resolution of the super hyperfine structure of the ESR spectrum of CTPO depends on interactions with other paramagnetic molecules. Changes in CTPO super hyperfine structure are characterized by a K parameter and the reduction in the K parameter is associated with a greater spin probe-spin probe and spin probe-molecular oxygen collisions. Irradiation of GNRs with an 808 nm laser resulted in a reduction in the super hyperfine structure of CPTO (Supplementary Fig. 2 and Supplementary Fig. 3), indicating the increased collision rate between CTPO molecules and/or between CTPO and residual molecular oxygen.


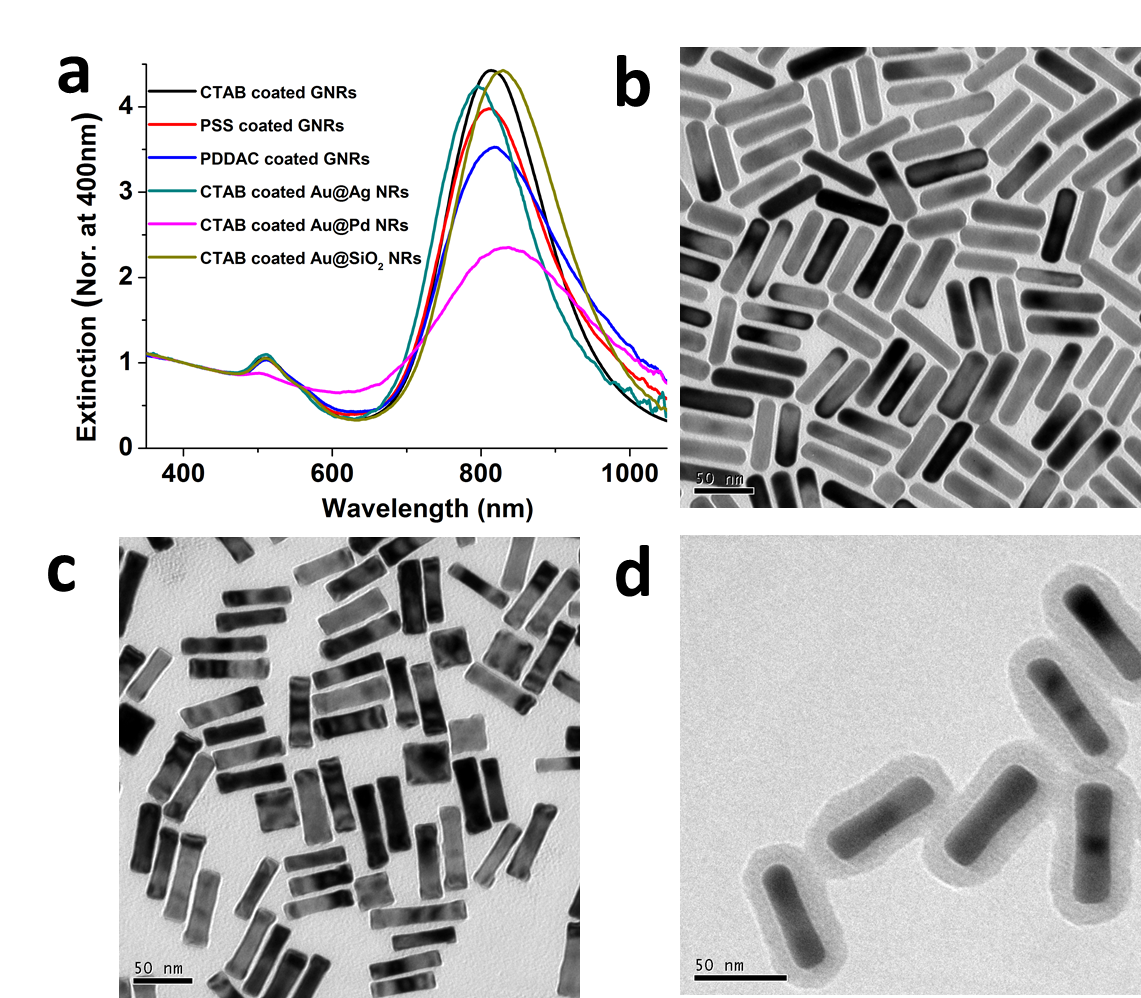


**Supplementary Figure 4.** (a) Normalized UV−vis−NIR spectra of nanomaterials with various coatings and components. Typical TEM images of (b) CTAB coated Au@Ag NRs, (c) CTAB coated Au@Pd NRs, and (d) CTAB coated Au@SiO_2_ NRs.

GNRs with six different surface alterations were synthesized and the conditions for surface alterations were selected to minimize effects on the wavelength (810 nm) needed for exciting the longitudinal SPR of the core GNR (Supplementary Fig. 4a). The types of surface alterations included coating with negatively charged PSS and positively charged PDDAC. Multilayer coating, achieved by first coating GNRs with PSS followed by coating with PDDAC, was an additional surface alteration. Another surface modification resulted from deposition of the metals, Ag and Pd, onto GNRs. Ag and Pd formed a uniform shell on the GNRs. In addition, ca. 10 nm thickness of SiO_2_ was coated onto GNRs forming a mesoporous shell.

The GNR particle concentration is estimated using inductively coupled plasma mass spectrometer (ICP-MS) and TEM as described in previous paper[^3^](#_ENREF_3). Generally, ICP-MS is employed to determine the total concentration of Au atoms in a given GNR suspension. According to the molar volume of a gold atom (10.2 cm^3^/mol), we obtain the total volume (V) of all GNRs in the suspension. From the TEM images, we measure the diameters and lengths of GNRs and then calculate the mean volume of a single GNR (V_0_) by assuming a cylindrical shape with two half-sphere end-caps. The GNR molar concentration in the suspension can be estimated as V/AV_0_, where A is Avogadro’s constant. The particle concentrations of other nanoparticles are all based on GNR particle.


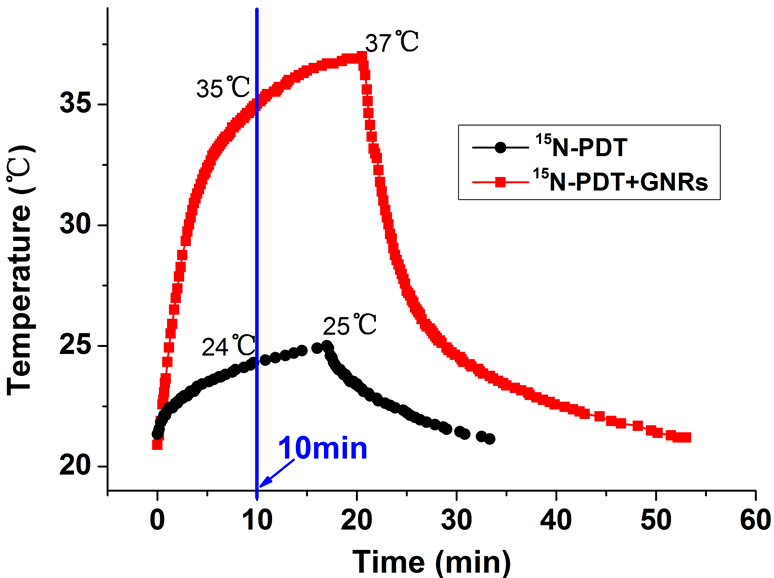


**Supplementary Figure 5.** Temperature changes for samples containing 100 μM ^15^N-PDT in the absence (black) or presence (red) of 2.4 nM (particle concentration) GNRs during and after irradiation with a 5 W 808 nm laser.


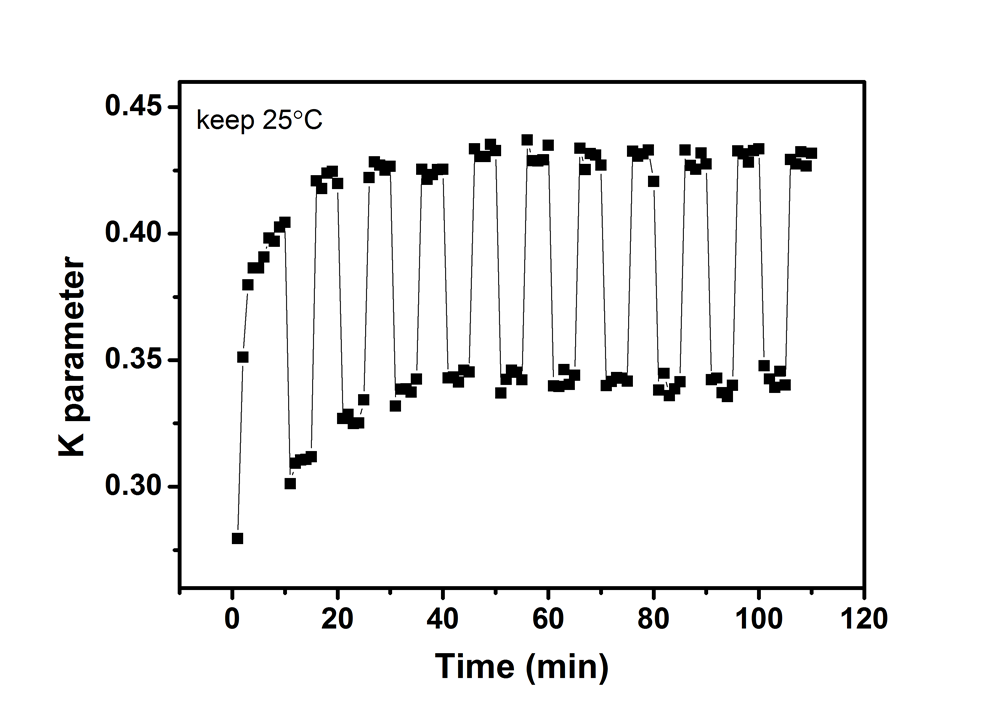


**Supplementary Figure 6.** Cyclicity of K parameter changes for 100 μM CTPO in the presence of 2.4 nM CTAB coated GNRs. Samples were contained in a TPX capillary purging with N_2_. Cyclical behavior is observed when repeatedly turning on/off the 5 W 808 nm laser for samples purged with N_2_. The temperature was maintained at 25 ˚C.


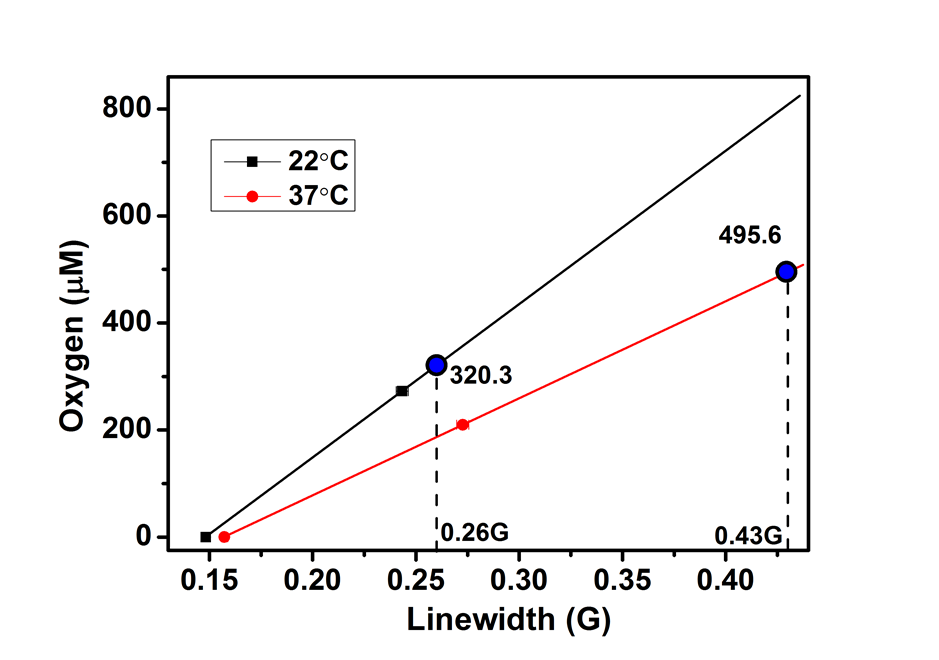


**Supplementary Figure 7.** The relationship between ^15^N-PDT’s LWs and the concentration of oxygen at 22 ˚C and 37 ˚C.


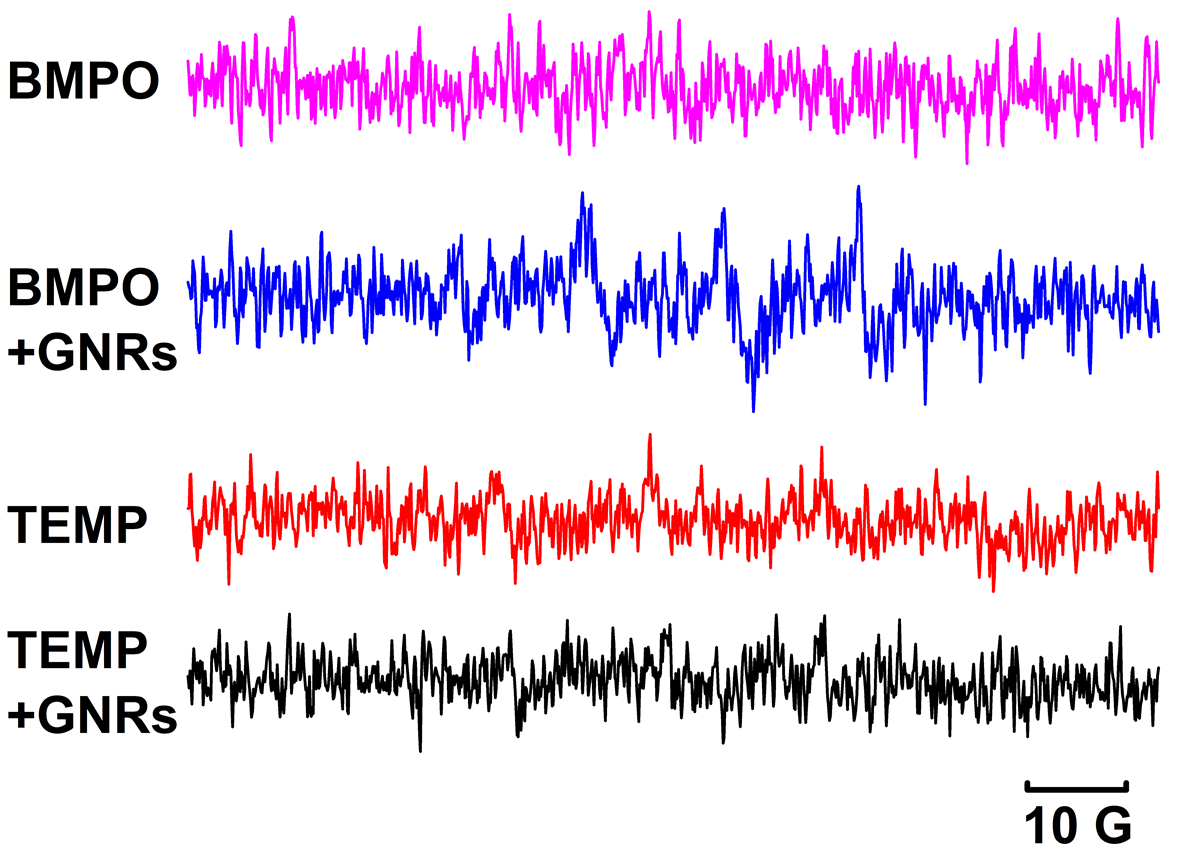


**Supplementary Figure 8.** ESR spectra for solutions with xenon lamp irradiation (λ > 420 nm) for 10 min. The concentrations are: [BMPO] = 25 mM, [TEMP] = 20 mM, and [GNRs] = 2.4 nM.


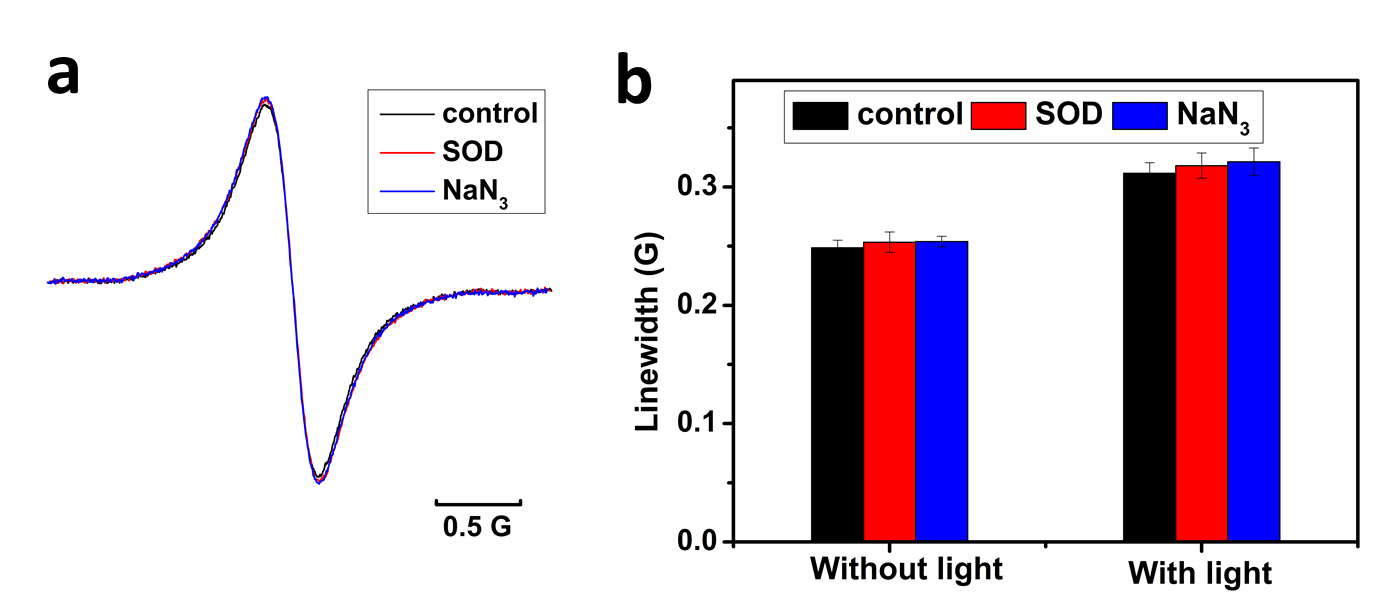


**Supplementary Figure 9.** (a) ESR spectra for solutions containing 100 μM ^15^N-PDT and 2.4 nM GNRs in the absence (control) and presence of 2U/mL SOD or 10 mM NaN_3_ with xenon lamp irradiation (λ > 420 nm) for 10 min. (b) LWs of ESR spectra.

Some of these ROS, e.g. superoxide radical anion and hydroperoxy radical, are paramagnetic and, if formed, could interact with ^15^N-PDT through spin exchange, resulting in an increased LW. Other potentially formed ROSs, such as singlet oxygen, are not paramagnetic but could conceivably cause increases in ^15^N-PDT’s LW through indirect mechanisms. Using ESR with spin trapping, we determined that neither superoxide radical nor singlet oxygen are formed during photoexcition of GNRs (Supplementary Fig. 8). We further examined the effects of superoxide dismutase (SOD)[^4^](#_ENREF_4), which catalyzes the decomposition of superoxide radical anion, and sodium azide[^5^](#_ENREF_5), which quenches singlet oxygen, on the effects of photoexcited GNRs (Supplementary Fig. 9). Addition of SOD or sodium azide had no effect on LW broadening induced by photoexcitation of GNRs. Therefore, neither superoxide radical anion nor singlet oxygen is involved in the effects of photoexcited GNRs on ^15^N-PDT’s LW.


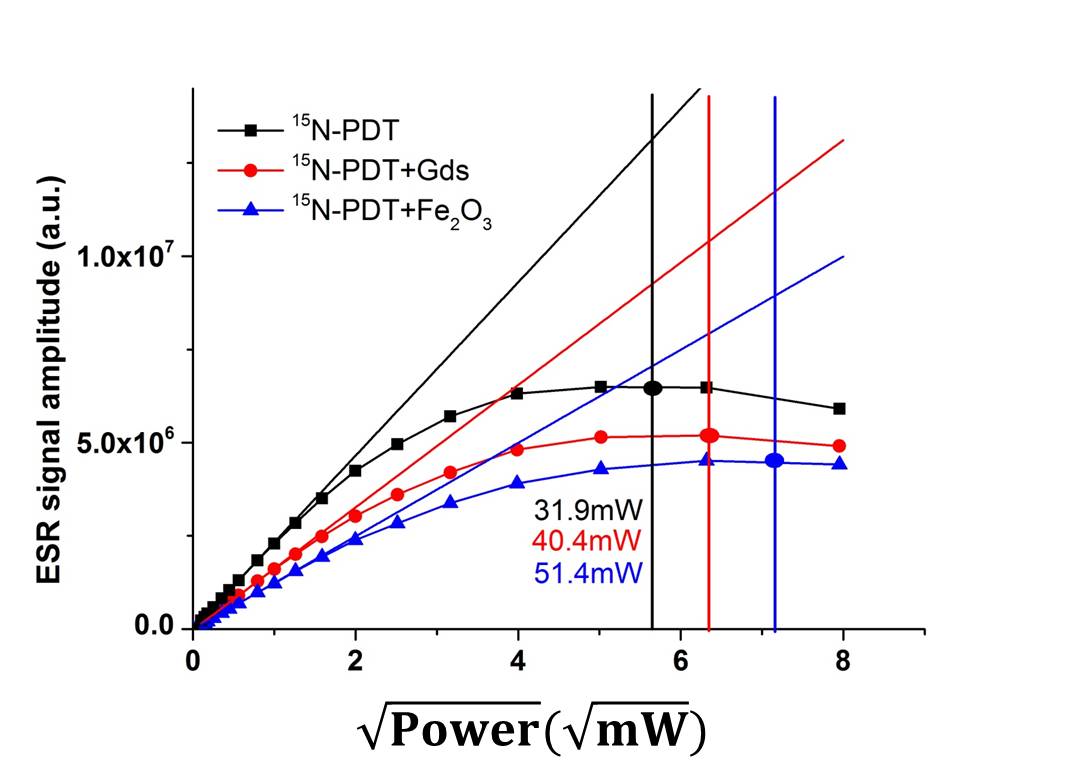


**Supplementary Figure** **10.** Power saturation curve for 100 μM ^15^N***-***PDT and none, 0.94 mg/mL Gds, 14 mg/mL Fe_2_O_3_ as noted. All are kept in 27 ˚C. A solid line (no saturation) extrapolating the initial slope shows the theoretical behavior in the absence of saturation.

The decrease on relaxation time seen in the presence of photoexcited GNRs was similar to that observed in the presence of Fe_2_O_3_ nanoparticles and Gds (Supplementary Fig. 10). This result illustrates the relative significance of the effect of photoexcited GNRs on the spin relaxation times of ^15^N***-***PDT.


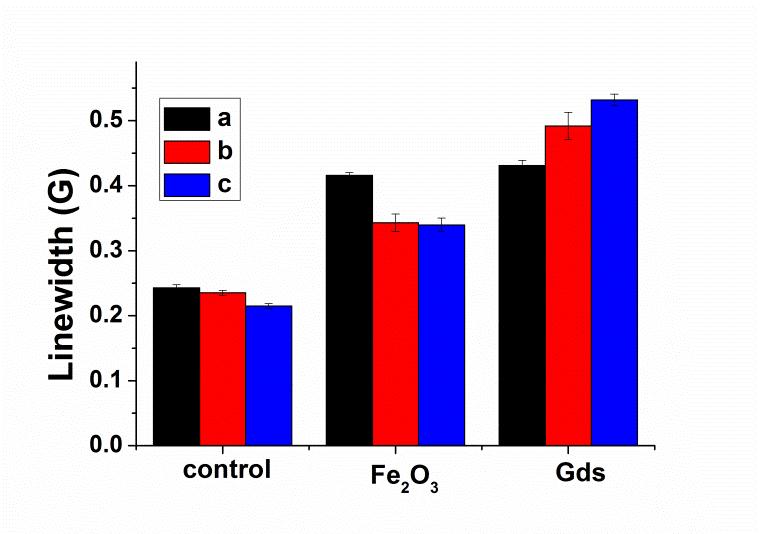


**Supplementary Figure** **11.** LW changes for paramagnetic contrast agents in solutions containing 100 μM ^15^N***-***PDT and none (control), Fe_2_O_3_ nanoparticles and Gadopentetate solution (Gds) with different viscosities (viscosity: a<b<c. Solutions of a, b, c, and d contain ca. 0%, 10%, and 19% glycerol, respectively) at 22˚C. The concentration of Fe_2_O_3_ nanoparticles is ca. 23 mg/mL, and Gds is 3.75 mg/mL.

We note that increasing viscosity also decrease the effects of superparamagnetic Fe_2_O_3_ nanoparticles on the LW of ^15^N-PDT. In contrast, the effect of gadopentetate on ^15^N-PDT’s LW increased with increasing viscosity (Supplementary Fig. 11). This observation indicates that a dipole-dipole interaction plays a predominate role in paramagnetic relaxation for this gadolinium-based MRI contrast agent. This is consistent with the results of Hyde and Sarna[^6^](#_ENREF_6), who determined that dipole-dipole interactions predominate in relaxation processes involving lanthanide ions, including gadolinium, and radicals.

**Reference**

1. Hyde, J. S. & Subczynski, W. K. Simulation of ESR spectra of the oxygen-sensitive spin-label probe CTPO. *J. Magn. Reson.* **56**, 125-130, (1984).
2. Lai, C.-S., Hopwood, L. E., Hyde, J. S. & Lukiewicz, S. ESR studies of O_2_ uptake by chinese hamster ovary cells during the cell cycle. *Proc. Natl. Acad. Sci.* **79**, 1166-1170, (1982).
3. Wen, T. *et al.* Copper ion assisted reshaping and etching of gold nanorods: mechanism studies and applications. *J. Phys. Chem. C* **117**, 25769-25777, (2013).
4. Salvemini, D., Riley, D. P. & Cuzzocrea, S. SOD mimetics are coming of age. *Nat. Rev. Drug Discovery* **1**, 367-374, (2002).
5. Lion, Y., Delmelle, M. & Van De Vorst, A. New method of detecting singlet oxygen production. *Nature* **263**, 442-443, (1976).
6. Hyde, J. S. & Sarna, T. Magnetic interactions between nitroxide free radicals and lanthanides or Cu^2+^ in liquids. *J. Chem. Phys.* **68**, 4439-4447, (1978).
